# Repeatability and relative validity of a quantitative food-frequency questionnaire among French adults

**DOI:** 10.3402/fnr.v56i0.18472

**Published:** 2012-10-30

**Authors:** Emmanuel Barrat, Nicolas Aubineau, Matthieu Maillot, Élodie Derbord, Pauline Barthes, Jean-François Lescuyer, Nathalie Boisseau, Sébastien L. Peltier

**Affiliations:** 1 Laboratoire Lescuyer, Department of Research, Aytré, France; 2 INSERM, UMR1062, Nutrition, Obesity and Risk of Thrombosis, Marseille, France; 3 INRA, UMR1260, Marseille, France; 4 Faculté de Médecine, Aix-Marseille University, Marseille, France; 5 Clermont Université, Université Blaise Pascal, EA 3533, Laboratoire des Adaptations Métaboliques à l'Exercice en conditions Physiologiques et Pathologiques (AME2P), BP 80026, F-63171 Aubière Cedex, France

**Keywords:** nutrient intake, diet record, relative validation, adult, France

## Abstract

**Background:**

A 50-item self-administered food frequency questionnaire (FFQ) was developed for French adults, to assess the intake of energy, 10 macronutrients, 11 vitamins, and 11 minerals, and to be used in the context of a medical consultation.

**Objective:**

To assess the repeatability and relative validity of this FFQ compared to a 7-day diet record (7-DR).

**Design:**

A total of 54 and 100 French adults were included in the repeatability and validation studies, respectively. Repeatability was assessed using two FFQs, the second carried out 3 weeks after the first. In the validation study, subjects first completed the FFQ, then the 7-DR the following week. Energy and nutrient intakes were compared using Pearson correlation. The degree of misclassification by the FFQ, compared to the 7-DR, was calculated by a contingency table of quintiles. Bland–Altman plots assessed the correlation between FFQ and 7-DR across the intake range.

**Results:**

Repeatability for intake, explored by Pearson correlation, was 0.62–0.90 (median: 0.81). Relative validity, as determined by Pearson correlation for the nutrient intake derived from the FFQ and 7-DR, was 0.36–0.80 (0.64). The FFQ tended to report higher fiber and micronutrient intake than 7-DR. Misclassification into opposite quintiles ranged 0–6% (1%), whereas classification into same or adjacent quintiles ranged 59–83% (74%). Bland–Altman plots showed good agreement for most nutrients across the range of intake.

**Conclusion:**

This new FFQ showed a high repeatability and good relative validity, and thanks to its short length, should be a useful tool for rapidly evaluating the nutrient intake of French adults.

Recent reports have shown that nutritional deficiencies are to be found even in industrialized countries, but particularly in certain populations, for example, women, children, and the elderly. The most studied examples are related to vitamin D, folate, vitamin B6, vitamin B12, calcium, iron, and iodine (1–3). The health impacts of these nutritional deficiencies are not fully understood. Although the clinical manifestations of profound nutritional deficiencies are well established (4), the health consequences of chronic low-grade deficiencies are still unclear. Further research is needed to achieve consensus, but it is clear that an adequate diet supplying a satisfactory amount of nutrients is essential for maintaining the normal functions of the body and thus prevent chronic disease (5–7).

Primary healthcare is vital in the prevention of chronic disease, promoting regular physical activity and good nutrition (8). General practitioners are playing a key role here, evaluating the risk of nutritional deficiencies, providing general advice on what is considered healthy dietary habits, and, if necessary, referring the patient to a competent health professional (i.e. dietician, nutritionist, or endocrinologist, for the construction of a diet plan, and/or psychiatrist for the resolution of any eating disorders). Dietary supplements are often proposed until healthy dietary habits are providing adequate nutrient intake, although the choice of which nutrient to be supplemented is generally subjective.

The need for specific tools to help the general practitioner evaluate each patient's risk of nutritional deficiencies is therefore of great importance. These tools must be inexpensive, quick and easy to use, reproducible, and validated. Several methods have been developed to evaluate individual dietary intake, for example, 24-h dietary recalls (24-HRs), diet records (DRs), or food frequency questionnaires (FFQ). FFQ seems the best suited for use by general practitioners since they do not require trained personnel and can be self-administered (9). In addition, since they first appeared, FFQs have been improved greatly to become semiquantitative, taking into account not only consumption frequency but also the portion size of a list of food items (generally between 100 and 200 items). However, FFQs have limitations such as an increased risk of bias and the need for the chosen items to be representative of a specific population's dietary habits. For these reasons, FFQs need to be validated, usually by comparing them to DRs or 24-HRs. Four FFQs have been developed and validated for the French population based on lists of 124 (10), 134 (11), 238 (12), and 240 (13) food items. Although the time required to complete these questionnaires is less than for a DR, it is still excessive, which limits their use among general practitioners.

The aim of this study was therefore to develop a shorter self-administered quantitative FFQ and assess its repeatability and validity compared to a 7-day diet record (7-DR).

## Materials and methods

### Development of the FFQ

#### Design of the questionnaire

The questionnaire used in this study was developed to evaluate daily energy, macronutrient, and micronutrient intake during a medical consultation. In order for it to be completed quickly, many items in the questionnaire were not specific foodstuffs, but food groups, thus reducing the number of items.

#### Definition of the items used in the questionnaire

There were 50 items in the questionnaire of which 27 were food group items (Table 1) and the others individual foodstuffs, for example, olive oil. The items were defined by a dietician (NA), based on his clinical experience of assessing French dietary habits, with the aim of evaluating intake of macronutrients, particularly fiber and polyunsaturated lipids, vitamins, and minerals.

**Table 1:** Food items, categorized into major food categories, included in the food frequency questionnaire

Major food categories	Food items
Fruit and vegetables	Raw vegetables
	Cooked vegetables
	Dried fruits
	Fruits
Proteins	Red meats
	White meats
	High-fat delicatessen, e.g. foie gras
	Low-fat delicatessen, e.g. ham
	Offal
	Eggs
	Fatty fish
	Semi fatty fish
	Lean fish
	Seafood
Cereals, grains, and potatoes	Bread (mostly white)
	Bread (mostly whole grain)
	Beans and other pulses
	Cereals, pasta, and rice
	Cold breakfast cereals
	Potatoes
Dairy	Cheese
	Creamy desserts and ice creams
	Other dairy products
	Soya milk
Oils and fat	Butter
	Margarine
	Sour cream
	Sunflower seed oil
	Colza oil
	Olive oil
	Corn oil
	Grape seed oil
	Groundnut oil
	Oil blend rich in omega 3
	Soya oil
	Walnut oil
Prepared foods	Cooked dishes
	French fries
	Fried & reconstituted products
	Pizza and other savory tarts
Beverages	Wine
	Other alcoholic beverages
	Coffee and tea
	Water
	Sugar sweetened beverages
Sugary foods	Chocolate
	Jam, marmalade, and honey
	Cakes and pastries
	Sugar
Condiments	Salt

First, the food groups consisted of foodstuff homogenous in terms of energy and their macronutrient content, for example, all fruits were combined into one group. Second, the intake of a food group needed to be easily evaluated by the subject and hence subgroups were created, if needed. For instance, the vegetable group was subdivided into ‘raw vegetables’ and ‘cooked vegetables’ to improve the accuracy of the participant's recall, since cooked and raw vegetables are usually eaten during different parts of a meal. Third, we chose to further estimate fiber and polyunsaturated lipid intake, so the initial groups of bread, fish, and oil were further divided. The bread group was subdivided into ‘mostly whole grain’ or ‘mostly white’ bread to provide a better estimation of fiber intake. To adequately estimate the intake of polyunsaturated fatty acids, the most frequently consumed oils were listed individually and the fish group was divided into three subgroups, according to their polyunsaturated fatty acid content.

The final list of items was ultimately validated by checking that all food groups contributing to at least 5% of the mean energy, macronutrient, vitamin, or mineral intake of the French population were represented in the 50 items of the FFQ, using data from the second national study of the individual food intake of French adults (14).

#### Nutrient profile of food items

Nutrient profiles of the 50 items were derived from the French food composition database (FFCD), drawn up by the French Data Centre on Food Quality (CIQUAL, last updated in 2008). Every individual food listed in the FFCD that corresponded to an item on the questionnaire (single food or food group) was included with the only exception being foods that are never consumed. For instance, the ‘white meat’ group included all the white meats listed on the FFCD food list except raw meat, as white meat is rarely, if ever, consumed raw. The energy, macronutrient (including fiber and cholesterol), and micronutrient (11 vitamins and 11 minerals, see Table 3 for details) content per 100 g of each of the 50 items was calculated as the average of the nutrient composition of each individual foodstuff included in the food group item, using Nutrilog software (v2.10d) on the latest CIQUAL food composition database.

#### Assessment of nutrient intake from the questionnaire

To complete the questionnaire, 15–20 min was necessary. Subjects were asked to base their estimation of food intake on the previous week. For each of the 50 items, subjects were asked about the portion size they usually consumed and the mean consumption frequency. The frequency was evaluated with a semi-open question since the participant had to tick and complete one of three boxes: ‘never or less than once a week’, ‘*x* time(s) a week’ and ‘*x* time(s) a day’. A typical portion size was estimated either via a multiple-choice portion scale in grams, adapted to the item, or via the number of portion per se (for fruits, eggs, dairy products, etc.). Participants were guided regarding usual portion size and weight in grams, for example, ‘1 tomato weighs 100 g=3 slices of aubergine=3 broccoli florets or 2 small endives’.

The amount of each food item consumed was estimated by multiplying the usual portion size by the mean consumption frequency. Nutrient intake of subjects was calculated by summing the product of each food item nutrient profile with the corresponding amount of food item consumed.

### 7-Day dietary record

The 7-DR was created by a dietician. It included information regarding the equivalence between portion size and weight in grams for each main food group and a table for recording food intake. For 7 consecutive days, the subject had to write down what and wherever possible the weight of all food and drink consumed both during meals (breakfast, lunch, and dinner) and between meals. For each meal (and inter-meal), a specific box was used for the intake of oil, butter, sugar, and salt.

The mean daily energy and nutrient intakes were calculated using Nutrilog software (v2.10d) and the CIQUAL food composition database. The box ‘sun exposition’ was not checked so that only vitamin D intake from food was taken into account.

### Subjects

This study was performed in accordance with the Helsinki Declaration of 1975, as revised in 2008.

#### Repeatability

A total of 68 healthy subjects, residing mainly in the South-West of France, were recruited from November 2011 to January 2012. The aims of the study were explained to them by a technician. The subjects completed the first FFQ and then the second FFQ 3 weeks later. The FFQs were simply completed at home by the subject and immediately returned by post. A data manager reviewed the completed questionnaires and contacted the subjects if necessary to provide missing information or clarify their responses. Fourteen subjects were excluded from the study: seven were excluded because they did not complete the second FFQ, two were excluded because of adherence to a special dietary regimen after the first FFQ, four because too much data was missing, and one male subject because, according to the second FFQ, his energy intake was 687 kcal per day (below the cutoff of 800 kcal per day). Thus, 54 subjects were included in the statistical analysis, whose characteristics are presented in Table 2.

**Table 2:** Age§, body mass index (BMI)§, gender, and place of residence of the subjects included in the repeatability and validation study

	Repeatability (*n*=54)	Validation (*n*=100)
Age (year)	37.1 (14.0)	37.5 (13.9)
BMI (kg/m^2^)	23.0 (3.5)	22.8 (3.1)
Gender (%)		
Men	24	29
Women	76	71
Place of residence (%)		
North-West	4	88
South-West	88	5
East	8	7

§ Expressed as mean (SD).

#### Validity

A total of 106 healthy subjects, mostly from the North-West of France, were recruited by a contract research organization (Biofortis, Nantes, France), from September 2010 to April 2011. The specificities of this study were explained by a technician who showed the subjects how to complete the 7-DR. Each subject was asked to first complete the FFQ based on the previous week, then, during the following 7 days, the food record. Thus, the two methods did not assess diet over the same time span. This was chosen because subjects would have been artificially aware of their dietary habits by completing the 7-DR the week before completing the FFQ. The FFQ and the 7-DR were completed at home by the subject and immediately returned by post. As before, the data was reviewed and the subject was contacted if necessary but this time a dietician also reviewed the data of the 7-DRs. At this stage, six subjects were excluded from the study: four because they did not estimate the quantity of their food intake in the 7-DR and two because too much information was missing in either the 7-DR or the FFQ. Therefore, 100 subjects were included in the statistical analysis, whose characteristics are presented in Table 2.

### Statistics

Statistical analysis was performed with SAS v9.1 (2009, SAS Institute) and GraphPad Prism v5.04 (2010, GraphPad software Inc.). The Gaussian distributions of nutrient intake as estimated from the FFQs or the 7-DR were tested using the non-parametric Kolmogorov–Smirnov test. All the variables not normally distributed were log10-transformed. Energy adjustment was performed using the residual method proposed by Willett and Stampfer (15).

#### Repeatability

The total nutrient intake as discerned from the two FFQs was statistically described by the mean±standard deviation (SD). The average nutrient intake as assessed from the second FFQ was also expressed as a percentage of the mean nutrient intake estimated from the first FFQ. A Student's paired t-test was used to assess whether nutrient intakes estimated with both FFQs were statistically different. The repeatability of the FFQ was estimated using the Pearson correlation coefficient, first as a crude estimate and then adjusted for total energy intake.

#### Validity

The average nutrient intake as assessed by the FFQ was also expressed as a percentage of the mean nutrient intake as estimated by the 7-DR. A Student's paired t-test was used to assess whether nutrient intakes when estimated using 7-DR and FFQ were statistically different.

Intake of the various nutrients estimated from the 7-DR was used as the reference to validate the performance of the FFQ assuming that the 7-DR represented actual intake. The concordance of nutrient intake from the FFQ and the 7-DR was estimated using the Pearson correlation coefficient, first as a crude estimate and then adjusted for total energy intake. For both nutrient intake assessment methods, subjects were classified based on quintile categories of energy and nutrient intake. The proportion of subjects classified into the same, same+adjacent, and extreme quintiles by the two methods were obtained from the crude variables. Bland–Altman plots on energy-adjusted values were used to evaluate agreement between the FFQ and the 7-DR. The mean differences between the two methods of assessment (FFQ and 7-DR) were plotted against the average estimation of the two methods. The 95% limit of agreement was calculated as the mean difference ±1.96 SD.

## Results

### Repeatability

Absolute daily intake of energy and nutrients was comparable between the two FFQs (Table 3) although most nutrients showed a slight decrease between FFQ1 and FFQ2 (median −5%). Pearson correlation coefficients estimating repeatability are shown in Table 3. For energy and macronutrients, crude correlation coefficients ranged from 0.62 (fiber) to 0.87 (cholesterol), with a median of 0.80. For micronutrients, crude correlation coefficients ranged from 0.67 (vitamin C) to 0.90 (cobalamin), with a median of 0.82. Energy-adjusted correlation coefficients ranged from 0.59 (fiber) to 0.89 (cobalamin), with a median of 0.79.

**Table 3:** Repeatability of the food frequency questionnaire (FFQ) shown by mean (SD) daily estimates of nutrients and Pearson correlation between the crude and energy-adjusted estimations derived from the first and second FFQs (*n*=54)

	Daily intakes			Pearson correlation coefficient	
	
	FFQ1	FFQ2	FFQ2 (% FFQ1)	Crude	Energy-adjusted
Energy, kcal$	1,814 (497)	1,721 (526)*	95	0.78	–
Protein, g	80.3 (24.4)	78.9 (27.6)	98	0.80	0.79
Carbohydrate, g	189 (53)	171 (55)*	91	0.67	0.70
Fat, g	77.9 (30.9)	76.4 (33.8)	98	0.81	0.71
MUFA, g	31.6 (14.7)	31.8 (17.0)	101	0.78	0.74
PUFA, g§	12.2 (7.1)	12.3 (8.3)	101	0.84	0.76
SFA, g	27.3 (10.7)	25.8 (11.2)	95	0.83	0.67
Cholesterol, mg	261 (116)	265 (122)	101	0.87	0.85
Fiber, g	22.1 (7.0)	19.4 (7.1)*	88	0.62	0.59
Vitamin A, µg§	417 (368)	421 (416)	101	0.86	0.76
Thiamine, mg§	1.3 (0.4)	1.3 (0.5)	96	0.77	0.77
Riboflavin, mg§	1.9 (0.8)	1.7 (0.8)*	93	0.89	0.87
Niacin, mg§	18.8 (6.6)	18.6 (7.6)	99	0.82	0.79
Pantothenic acid, mg§	6.0 (2.2)	5.6 (2.2)*	93	0.86	0.83
Pyridoxine, mg	2.0 (0.7)	1.9 (0.7)	95	0.85	0.83
Folic acid, µg	357 (122)	316 (115)*	88	0.77	0.78
Cobalamin, µg§	5.1 (3.1)	5.2 (3.5)	103	0.90	0.89
Vitamin C, mg	165 (71.2)	140 (59.6)*	85	0.67	0.68
Vitamin D, µg§	2.8 (1.9)	3.1 (2.5)	111	0.80	0.68
Vitamin E, mg§	12.7 (6.8)	11.9 (7.0)	94	0.83	0.79
Calcium, mg§	710 (319)	648 (345)*	91	0.83	0.80
Iron, mg	13.5 (4.2)	12.6 (4.3)*	93	0.84	0.83
Magnesium, mg	278 (78.7)	255 (80.9)*	92	0.80	0.80
Zinc, mg	10.1 (3.1)	9.6 (3.1)	95	0.80	0.79
Selenium, µg	51.6 (20.2)	52.3 (23.8)	101	0.85	0.84
Phosphorus, mg	1,222 (366)	1,175 (407)	96	0.82	0.80
Manganese, mg	3.4 (1.0)	2.9 (1.1)*	86	0.72	0.72
Potassium, mg	3,046 (894)	2,812 (897)*	92	0.74	0.73
Iodine, µg	111 (44)	107 (49)	96	0.81	0.76
Copper, mg§	1.6 (0.7)	1.4 (0.7)*	89	0.85	0.84
Sodium, mg	2,342 (722)	2,230 (709)	95	0.68	0.67

$ Daily intakes are expressed as mean (SD).

§ Nutrient intakes were transformed (log_10_) to improve normality.

* *p*<0.05 FFQ2 compared to FFQ1 (paired t-test).

### Validity

Mean macronutrient intake estimated using the FFQ was not statistically different from that estimated using the 7-DR (Table 4) except for fiber intake that was overestimated (+32%) by the FFQ. On the contrary, mean micronutrient intake was significantly higher when estimated by the FFQ, compared to the 7-DR, except for niacin, cobalamin, calcium, sodium, phosphorous, and iodine. Mean overestimations ranged from +6% (vitamin A) to +74% (vitamin C), with a median of +9%.

**Table 4:** Validation of the food frequency questionnaire (FFQ) shown by mean (SD) daily estimates of nutrients and Pearson correlation between crude and energy-adjusted estimations derived from the 7-day dietary record (7-DR) and the FFQ (*n*=100)

	Daily intake			Pearson correlation coefficient	
	
	7-DR	FFQ	FFQ (% 7-DR)	Crude	Energy-adjusted
Energy, kcal	1,571 (368)$	1,558 (445)	99	0.77	–
Protein, g§	68.5 (15.2)	68.2 (18.5)	100	0.72	0.69
Carbohydrate, g	166 (43)	160 (51)	96	0.76	0.75
Fat, g	64.7 (20.4)	66.2 (23.9)	102	0.75	0.62
MUFA, g§	24.6 (8.9)	26 (10.5)	106	0.78	0.72
PUFA, g§	8.7 (4.5)	9.1 (5.1)	105	0.75	0.58
SFA, g	24.6 (8.7)	24.9 (9.8)	101	0.68	0.60
Cholesterol, mg	247 (90)	249 (106)	101	0.57	0.48
Fiber, g§	14.5 (5.3)	19.1 (7.6)*	132	0.73	0.71
Vitamin A, µg§	366 (462)	386 (455)*	106	0.57	0.19
Thiamine, mg	1 (0.3)	1.1 (0.4)*	110	0.54	0.50
Riboflavin, mg	1.4 (0.5)	1.6 (0.5)*	114	0.71	0.72
Niacin, mg§	14.7 (4.2)	15.5 (4.9)	105	0.57	0.49
Pantothenic acid, mg	4.2 (1.2)	5.1 (1.6)*	121	0.60	0.56
Pyridoxine, mg	1.4 (0.4)	1.7 (0.5)*	121	0.61	0.59
Folic acid, µg	234 (81)	315 (110)*	134	0.59	0.63
Cobalamin, µg§	4.9 (5)	4.5 (2.8)	92	0.80	0.46
Vitamin C, mg§	84.8 (42)	147.7 (69)*	174	0.42	0.56
Vitamin D, µg§	2.5 (1.5)	2.9 (1.8)*	116	0.55	0.50
Vitamin E, mg§	8.8 (5.4)	10.1 (5.2)*	115	0.75	0.57
Calcium, mg	644 (202)	636 (218)	99	0.65	0.65
Iron, mg	9.9 (3)	11.7 (3.6)*	118	0.58	0.51
Magnesium, mg	216 (54)	240 (69)*	111	0.67	0.67
Zinc, mg	7.3 (2.1)	8.5 (2.5)*	116	0.60	0.44
Selenium, µg§	39.8 (18.6)	44.2 (14.2)*	111	0.54	0.50
Phosphorus, mg	1,051 (244)	1,064 (285)	101	0.68	0.60
Manganese, mg	2 (0.8)	2.8 (1.0)*	140	0.56	0.48
Potassium, mg	2,483 (636)	2,702 (811)*	109	0.64	0.64
Iodine, µg	101 (45)	102 (31)	101	0.45	0.55
Copper, mg§	1 (0.5)	1.3 (0.5)*	130	0.36	0.19
Sodium, mg	2,006 (610)	2,057 (765)	103	0.71	0.56

$ Daily intakes are expressed as mean (SD).

§ Nutrient intakes were transformed (log_10_) to improve normality.

* *p*<0.05 FFQ compared to 7-DR (paired t-test).

Crude and adjusted Pearson's correlation coefficients between the 7-DR and the FFQ are shown in Table 4. For macronutrient intake, correlation coefficients ranged from 0.57 (cholesterol) to 0.78 (monounsaturated fatty acids), with a median of 0.75. For micronutrients, correlation coefficients ranged from 0.36 (copper) to 0.80 (cobalamin), with a median of 0.59. After adjustment for energy intake, Pearson's correlation coefficients were almost comparable to those before adjustment, with the exception of vitamin A (*r*_adjusted_=0.19 vs. *r*_crude_=0.57) and cobalamin (*r*_adjusted_=0.46 vs. *r*_crude_=0.80).

When the macronutrient intake was categorized into quintiles (Table 5), the agreement rates for same or adjacent quintile classifications ranged from 72% (saturated fatty acids) to 83% (fibers). Complete misclassification, in opposite quintiles, ranged from 0% (protein, carbohydrate, monounsaturated fatty acids, and fibers) to 4% (saturated fatty acids and cholesterol). When micronutrients were considered, the agreement rates for same or adjacent quintile classifications ranged from 59% (vitamin A) to 81% (potassium). Misclassification of micronutrients into opposite quintiles ranged from 0% (riboflavin, magnesium, zinc, and potassium) to 6% (copper).

**Table 5:** Cross-classification of daily energy-adjusted nutrient intake derived from the food frequency questionnaire (FFQ) and the 7-day dietary record (7-DR), expressed as percentage of subjects classified in the same, same and adjacent, or extreme quintiles (*n*=100)

Nutrient (unit/day)	Same quintile	Same and adjacent quintile	Extreme quintiles
	% of subjects		
Protein, g§	39	80	0
Carbohydrate, g	44	80	0
Fat, g	39	75	1
MUFA, g§	41	77	0
PUFA, g§	33	78	3
SFA, g	39	72	1
Cholesterol, mg	38	72	4
Fiber, g§	40	83	0
Vitamin A, µg§	25	59	5
Thiamine, mg	35	74	1
Riboflavin, mg	36	78	0
Niacin, mg§	27	67	1
Pantothenic acid, mg	37	73	4
Pyridoxine, mg	40	75	2
Folic acid, µg	42	74	2
Cobalamin, µg§	39	72	4
Vitamin C, mg§	31	73	2
Vitamin D, µg§	31	69	5
Vitamin E, mg§	40	77	3
Calcium, mg	34	75	1
Iron, mg	35	68	1
Magnesium, mg	36	79	0
Zinc, mg	41	65	0
Selenium, µg§	27	65	1
Phosphorus, mg	33	77	1
Manganese, mg	39	73	2
Potassium, mg	38	81	0
Iodine, µg	39	75	1
Copper, mg§	30	68	6
Sodium, mg	40	73	0

§ Nutrient intakes were transformed (log_10_) to improve normality.

**Fig. 1:**
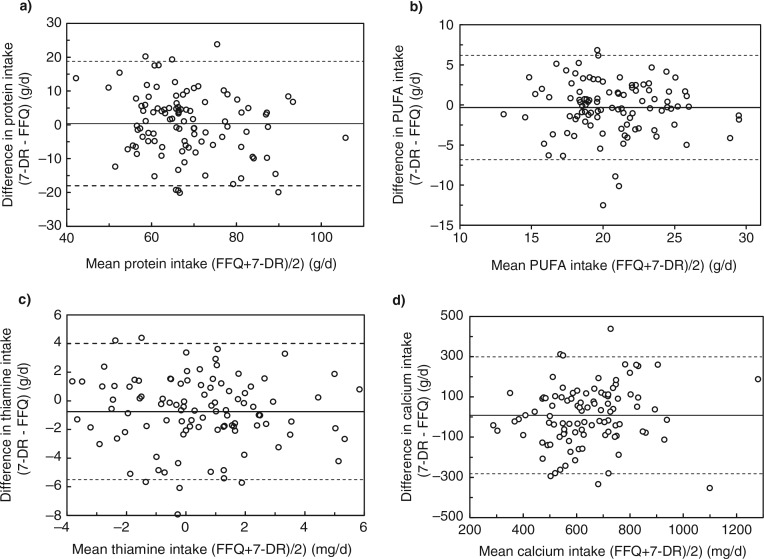
Bland–Altman plots representing the difference in the daily intake of energy-adjusted macronutrients and micronutrients intake derived from the food frequency questionnaire (FFQ) and the 7-day dietary record (7-DR) plotted against the corresponding mean energy-adjusted daily intakes derived from the two methods. Solid lines represent mean difference, and dashed lines show lower and upper 95% limits of agreement (mean±1.96 SD).

The Bland–Altman plot analysis graphs (Fig. 1) show agreement between the two methods of estimation, across the range of intake. First, for most macro- and micronutrients, the mean difference between methods was near zero for all levels of intake, except for fibers, folic acid, and vitamin C. Those nutrients were systematically overestimated by the FFQ, across the range of intake, which was consistent with the results in Table 4. Second, the percentage of plots that were outside the limits of agreement ranged from 3% (sodium and niacin) to 7% (protein, vitamin A, zinc, and copper), with a median value of 5%, which is theoretically the percentage of values outside the mean±1.96 SD. Finally, for most macro- and micronutrients, the agreement did not differ for low intake compared with high intake with the exception of vitamin E, selenium, and Iodine. The FFQ tended more often to overestimate vitamin E, selenium, and iodine when intake was low and to underestimate iodine when intake was high.

## Discussion

The aim of this study was to evaluate the repeatability and relative validity of a new FFQ on French healthy subjects. This FFQ was designed to rapidly evaluate daily macro- and micronutrient intake, based on 50 items, half of which were food groups. Although the use of food group items (rather than individual foodstuffs) could increase the risk of bias, the overall results indicate high repeatability (median correlation coefficient>0.80) and good relative validity (median correlation coefficient>0.60).

### FFQ structure

As the questionnaire was initially developed to be used during a medical consultation, we chose to limit the number of items to reduce the time needed to complete the questionnaire. Half of the 50 items in our FFQ were therefore food groups, instead of single foods. The number of food items listed in FFQs varies widely. In their review, Cade et al. (16) found that the number of items in FFQs published from 1980 and 1999 ranged from 5 to 350 items, with a median of 79 items. Several recent European FFQs contain more than 100 items (10, 13, 17–20). However, according to Willett (9), there is a rapidly decreasing marginal gain in information obtained with increasingly detailed questionnaires. With regards to the repeatability and relative validity of our FFQ, it appears that the 50 items chosen were sufficient to describe the nutrient intake of a population.

### Repeatability

#### Time frame

We chose a 3-week period between the two FFQs, to avoid major shifts in dietary habits and minimize loss of subjects. Nonetheless, two subjects, wishing to lose weight, reported changes in their dietary habits between the two questionnaires and had to be excluded.

#### Underestimation with FFQ2

As other authors have observed (10, 12, 17–19, 21–25), nutrient intake derived from a second FFQ is usually below that of the first: in our study the median difference in mean intakes was −5% (Table 3). This was shown in particular for fiber, folic acid, vitamin C, manganese, and copper, for which mean intake from the second FFQ was between 10 and 15% below that from the first FFQ. This effect could be due, in part, to a learning effect if we consider that the responses given in the second FFQ more accurately reflected real dietary habits. As we could not compare the actual completion of the FFQs with the 7-DR, it is impossible to know which FFQ is more valid. Nonetheless, several authors found their second FFQ, which indicated reduced nutrient intake, was more valid than the first when compared to 24-h recall (10, 18, 24), or DR (22, 25), confirming the possibility of a learning effect.

#### Comparison with other studies

The crude or energy-adjusted Pearson correlation coefficients observed during the current study (median of crude coefficient: 0.81) compared favorably with other short-term European repeatability studies (two FFQs within 3 months) tested on adults (17, 26, 27) and were close to those described in an Italian FFQ (median of crude coefficient: 0.85) (19). Compared with the other French FFQs, the repeatability of the present FFQ was higher as their median correlation coefficients were 0.55 (10), 0.66 (12), and 0.67 (13). It should be noted that the repeatability of these FFQs were tested at a 1-year interval, which increased the risk of real changes in dietary habits and may have reduced the correlation coefficient.

### Validity

#### Reference method

The reference method used in this study for determining validity was a 7-DR, another method of assessing diet. Although 7-DRs present bias, they are considered as reference methods better able to validate FFQs, compared to 24-h recall, because they do not share with FFQs the same sources of error (e.g. reliance upon memory, conceptualization of portion sizes and distortion of reported diet) (16).

#### Subjects and study population

Our validation and repeatability studies were performed on adult volunteers, who tend to provide more accurate responses to questionnaires. In addition, they were mostly females residing in the West of France (Table 2), giving us no guarantee that our study population was representative of the general adult population residing in the East of France. This could be considered as a limitation of this study.

We included 54 subjects in the repeatability study and 100 subjects in the validation study. An appropriately powered study, when Bland–Altman plots are used, should include at least 50 but preferably 100 or more subjects. For the correlation coefficient, when 14 days of dietary information are obtained, no more than 100 should be sufficient (16). Taken together, these recommendations indicate that our study was moderately powered, as is often the case (19, 20, 23), although a number of recent studies have been less powered (18, 28, 29).

#### Comparison with other studies

To our knowledge, this is the first French FFQ to report validation of the intake of thiamine, niacin, pantothenic acid, pyridoxine, cobalamin, vitamin D, magnesium, zinc, selenium, phosphorus, manganese, iodine, copper, and sodium. With regard to macronutrients and other micronutrients, the present FFQ demonstrated good relative validity compared to the other French FFQs (10–13). With the exception of vitamin C, all the macro- and micronutrient crude correlation coefficients reported here were above those reported in the other four French studies. When considering agreement between classifications by intake level, the present FFQ showed similar or higher agreement compared to the other three French FFQs that reported it (10–12).

Since the four other FFQs comprised 124–240 items, the results of the present FFQ—containing only 50 items—are highly satisfactory. As the FFQ was used only after the DR, we can rule out a learning effect as the cause of the increased relative validity. However, other factors might partly explain this higher relative validity, that is, a higher diversity of intake of the population, a higher number of days of record for the reference method, a higher number of subjects, and a higher educational level. Although this last factor cannot be ruled out since our population's educational level was not evaluated, it is unlikely that the higher relative validity of the present FFQ be explained by the other factors cited. Indeed, one French FFQ was validated among 108 female hospital staff with a probable high educational level, compared to 12 days of recall (12). Another FFQ was validated among a diversified population, also compared to 12 days of recall (13).

Pearson correlation coefficients observed in this study also compared favorably with those reported in most European studies (17, 30–32), with the exception of two questionnaires recently validated in Italy (19) and Spain (20), showing median correlation coefficients of 0.78 and 0.82, respectively. The higher correlation coefficients could theoretically be explained by the higher number (189 and 113 items, respectively) and the choice of food items, which could better represent dietary intake. The authors of these studies reported biases in their studies, that is, the use of the same serving size tool in both methods of diet estimation and a quick list used for the 24-HR recall generated from the food items reported in the FFQ (19) or subjects who have been aware of their dietary pattern due to health-related dietary recommendation (20).

#### Principal limitations

Although not noted with other macronutrients, the mean daily fiber and micronutrient (particularly vitamin C) intake obtained from the FFQ was significantly higher than that derived from the 7-DR. Therefore, one should be aware that the use of this FFQ will minimize identification of the risk of nutritional deficiency. This trend has been noted in some other validation studies using either DRs (21, 30) or 24-h recall (10, 19, 20), albeit not in all studies (29). One reason for this difference could be an underestimation by the 7-DR, and/or an overestimation by the FFQ. Overestimation by the FFQ could be due to inaccurate subject reporting of frequency and/or mean portion size. Over-reporting of fruit and vegetable consumption by subjects seeking social approval is a common bias (33). Since fruit and vegetables are rich in micronutrients (particularly vitamin C) and fibers and poor in energy and macronutrients, overreporting of fruit and vegetables could account in part for the over-estimation of fibers, vitamins and minerals and not of energy and macronutrients. Another reason for this overestimation may lie in the use of food group items, instead of individual foodstuffs, especially for fruits and vegetables. It is indeed possible that a certain monotony in an individual's dietary habits, eating only one food (e.g. apple) from within a food group itemized in our FFQ (e.g. fruits) could have induced an overestimation of micronutrients but not macronutrients.

The present FFQ also showed a disappointing evaluation of copper and vitamin A intake, when compared to a 7-DR. The principal source of vitamin A identified by the FFQ in the validation study, as well as in the French population (14), is offal, a food group that contains very high amounts of vitamin A but one that is not generally consumed regularly. van Liere reported that the lowest correlations were found for foods that are not consumed regularly (12). In the present study, five out of the 100 subjects reported ‘never or less than once a month’ for the consumption of offal in the FFQ but reported consumption of this food group during the seven following days in the diet report. This explains, in part, the low correlation for vitamin A. It is probable that 7 days of diet report are not sufficient to best describe the mean intake of vitamin A. Therefore, vitamin A intake derived from the FFQ in its present form should be taken with precaution, especially for occasional eaters of offal. In the future, the FFQ that we develop will distinguish the frequency between the non-eater and the occasional eater (less than once a week). The main sources of copper were breakfast cereals, fruits, and vegetables. Correlation with 7-DR was below 0.45 and lower after energy-adjustment (*r*=0.19), and the reason is uncertain. Therefore, care should be taken when interpreting copper intake as evaluated by our FFQ.

In conclusion, this 50-item self-administered FFQ has demonstrated, despite the reduced number of items, high repeatability and good relative validity. The evaluation of energy and macronutrients intake showed a high level of relative validity. For the first time, a French FFQ was able to determine dietary intake of all vitamins and minerals, with moderate to high relative validity. Like most of the FFQs, a tendency to overestimate dietary intake compared to DR was observed, meaning an underestimation of the risk of deficiency. Furthermore, it has been pointed out that the evaluation of vitamin A and copper intake presented a higher risk of error. Although it cannot replace an in-depth diet analysis, this new FFQ should be a useful instrument for any health practitioner, thanks to the short time needed to complete it.
